# Inhibitory Effect of KP-A038 on Osteoclastogenesis and Inflammatory Bone Loss Is Associated With Downregulation of Blimp1

**DOI:** 10.3389/fphar.2019.00367

**Published:** 2019-04-10

**Authors:** Hye Jung Ihn, Taeho Lee, Doohyun Lee, Jong-Sup Bae, Sang-Hyun Kim, Il Ho Jang, Yong Chul Bae, Hong-In Shin, Eui Kyun Park

**Affiliations:** ^1^Institute for Hard Tissue and Bio-tooth Regeneration, Kyungpook National University, Daegu, South Korea; ^2^College of Pharmacy, Research Institute of Pharmaceutical Sciences, Kyungpook National University, Daegu, South Korea; ^3^Department of Pharmacology, School of Medicine, Kyungpook National University, Daegu, South Korea; ^4^Department of Oral Biochemistry and Molecular Biology, School of Dentistry, Pusan National University, Yangsan, South Korea; ^5^Department of Oral Anatomy and Neurobiology, School of Dentistry, Kyungpook National University, Daegu, South Korea; ^6^Department of Oral Pathology and Regenerative Medicine, School of Dentistry, Institute for Hard Tissue and Bio-tooth Regeneration, Kyungpook National University, Daegu, South Korea

**Keywords:** imidazole, KP-A038, osteoclast, differentiation, bone resorption

## Abstract

Excessive osteoclastic activity results in pathological bone resorptive diseases, such as osteoporosis, periodontitis, and rheumatoid arthritis. As imidazole-containing compounds possess extensive therapeutic potential for the management of diverse diseases, we synthesized a series of imidazole derivatives and investigated their effects on osteoclast differentiation and function. In the present study, we found that a novel imidazole derivative, KP-A038, suppressed receptor activator of nuclear factor-κB ligand (RANKL)-mediated osteoclastogenesis and bone-resorbing activity *in vitro* and attenuated lipopolysaccharide (LPS)-induced bone destruction *in vivo*. KP-A038 significantly inhibited the induction of nuclear factor of activated T-cells, cytoplasmic 1 (NFATc1) and the expression of its target genes, including tartrate-resistant acid phosphatase (*Acp5*), cathepsin K (*Ctsk*), dendritic cell-specific transmembrane protein (*Dcstamp*), and matrix metallopeptidase 9 (*Mmp9*). KP-A038 upregulated the expression of negative regulators of osteoclast differentiation, such as interferon regulatory factor-8 (*Irf8*) and B-cell lymphoma 6 (*Bcl6*). Consistently, KP-A038 downregulated the expression of B lymphocyte-induced maturation protein-1 (Blimp1 encoded by *Prdm1*), a repressor for *Irf8* and *Bcl6*. Moreover, administration of KP-A038 reduced LPS-induced bone erosion by suppressing osteoclast formation *in vivo*. Thus, our findings suggest that KP-A038 may serve as an effective therapeutic agent for the treatment and/or prevention of bone loss in pathological bone diseases, including osteoporosis and periodontitis.

## Introduction

Bone remodeling comprises of resorption of aged or damaged tissue by osteoclasts and replacement of new bone by osteoblasts, which is delicately regulated in a steady physiological state. This process occurs continuously to maintain skeletal integrity and strength and mineral homeostasis. Enhanced osteoclast differentiation and resorbing activity lead to loss of bone mass and architectural deterioration of bone tissue, which is mainly responsible for osteolytic bone diseases, including osteoporosis, periodontitis, and rheumatoid arthritis (Manolagas and Jilka, 1995; Sato and Takayanagi, 2006; Bartold et al., 2010). Therefore, various studies have focused on the development of novel treatments targeting osteoclast formation and activation to prevent and treat osteolytic lesions.

Osteoclasts, the only cells capable of degrading the bone matrix, are formed through multiple steps, including proliferation, differentiation of monocyte/macrophage lineage progenitors into mononuclear preosteoclasts, fusion of preosteoclasts, and activation to break down bone (Chambers, 2000; Teitelbaum, 2000). Mature osteoclasts exhibit unique morphological characteristics, including a ruffled border membrane and an actin ring structure (Teitelbaum, 2011). Osteoclast differentiation and function are governed by two essential factors: macrophage-colony stimulating factor (M-CSF), required for the survival and proliferation of progenitor cells, and receptor activator of nuclear factor-κB ligand (RANKL), primarily involved in differentiation to osteoclasts (Feng and Teitelbaum, 2013). Upon binding of RANKL to its receptor RANK, intracellular signaling pathways, such as mitogen-activated protein kinases (MAPKs) and nuclear factor-kappa B (NF-κB), are activated, which eventually lead to the induction of nuclear factor of activated T-cells cytoplasmic 1 (NFATc1), a key regulator of osteoclast differentiation (Wong et al., 1998; Lee and Kim, 2003). The transcriptional activity of NFATc1 is repressed by several negative regulators, such as interferon regulatory factor-8 (IRF-8), v-Maf musculoaponeurotic fibrosarcoma oncogene family member protein B (MafB), and B-cell lymphoma 6 (Bcl6) (Kim et al., 2007; Zhao et al., 2009; Park-Min et al., 2013). During osteoclast differentiation, RANKL-induced B lymphocyte-induced maturation protein-1 (Blimp1, encoded by *Prdm1*) upregulation acts as a transcriptional repressor of these anti-osteoclastogenic transcription factors (Nishikawa et al., 2010).

Imidazole is a heterocyclic ring compound with molecular formula C_3_H_4_N_2_ and is a major constituent of various biological molecules, such as histidine, vitamin B12, and biotin (De Luca, 2006). Imidazole derivatives have been reported to exhibit a broad spectrum of biological and pharmacological effects, including anti-inflammatory, antiviral, antitumor, antifungal, and antimycobacterial activity, and numerous commercial drugs, like cimetidine, azithromycin, and metronidazole, contain imidazole nucleus in their structure (Fukui et al., 1982; el-Feky and Abd el-Samii, 1995; Johnson et al., 1999; Banfi et al., 2006). Due to these favorable and beneficial activities, studies have focused on the development of imidazole-based drugs in the pharmaceutical field (De Luca, 2006). Previous studies related to bone metabolism have revealed that imidazole and its analogs could suppress bone resorption, and a substituted imidazole, 4-nitroimidazole derivative, could inhibit RANKL-mediated osteoclast differentiation (Heersche and Jez, 1981; Chen et al., 2006). In addition, zoledronic acid, a potent third-generation bisphosphonate, contains an imidazole ring in the side chain (Paiva-Fonseca et al., 2014). However, it can induce unwanted adverse effects ranging from common to rare (Lambrinoudaki et al., 2008).

In order to develop a novel antiresorptive agent, we synthesized a series of imidazole derivatives and investigated their effects on osteoclastogenesis and bone-resorbing activity. In the present study, we demonstrated that KP-A038 significantly suppressed RANKL-mediated osteoclastogenesis *in vitro* and reduced lipopolysaccharide (LPS)-induced inflammatory bone erosion *in vivo*.

## Materials and Methods

### Antibodies and Reagents

**FIGURE 1:**
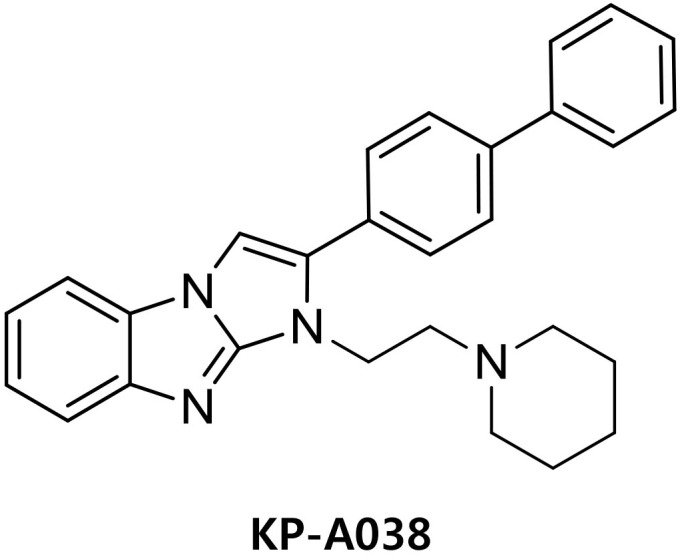
Chemical structures of KP-A038.

Antibodies against phospho-p38, phospho-JNK, phospho-ERK, and phospho-IκBα were purchased from Cell Signaling Technology (Danvers, MA, United States). Anti-NFATc1 antibody was purchased from BD Pharmingen^TM^ (San Diego, CA, United States), and antibody against IRF8 was obtained from Santa Cruz Biotechnology. Recombinant M-CSF and RANKL were obtained from R&D Systems (Minneapolis, MN, United States). Fetal bovine serum (FBS) and α-minimum essential medium (α-MEM) were obtained from Gibco BRL (Grand Island, NY, United States). KP-A038 is the imidazobenzimidazole compound of in-house chemical library and the chemical name of KP-A038 is (2-([1,1′-biphenyl]-4-yl)-1-(2-(piperidin-1-yl)ethyl)-1*H*-benzo[*d*]imidazo[1,2-*a*]imidazole (Figure 1). KP-A038 was synthesized as described previously (Supplementary Figure S1; Kim et al., 2014), and dissolved in DMSO for further experiments.

### *In vitro* Osteoclast Differentiation

Mouse bone marrow cells isolated from the tibiae and femora of 6–8 week-old male C57/B6L mice (Dae Han Bio Link Co., LTD., Chungbuk, South Korea) were incubated in α-MEM supplemented with 10% FBS (Ihn et al., 2017; Yoon et al., 2018). After 24 h, non-adherent cells were collected and cultured in α-MEM containing 10% FBS and M-CSF (30 ng/mL) for 3 days to generate bone marrow-derived macrophages (BMMs). BMMs were plated in 96-well plates and incubated in osteoclast-inducing media containing 20 ng/mL RANKL and 10 ng/mL M-CSF with or without various concentrations of KP-A038. The media were changed every 2 days until mature osteoclasts were formed. The cells were fixed in 4% paraformaldehyde, and formation of osteoclasts was determined using an Acid Phosphatase, Leukocyte (TRAP) staining kit (Sigma-Aldrich, St. Louis, MO, United States). TRAP-positive multinucleated cells (MNCs) with more than three nuclei were scored as osteoclast-like cells.

### Cell Viability Assay

The cytotoxic effect of KP-A038 on cell viability was determined using Cell Counting Kit-8 (CCK-8; Dojindo Molecular Technologies Inc., Rockville, MD, United States) following the manufacturer’s instructions. BMMs seeded in 96-well plates (3 × 10^3^ cells/well) were cultured in α-MEM containing 10% FBS and M-CSF (10 ng/mL) in the presence or absence of various doses of KP-A038 for 3 days. The media were replaced with fresh medium containing 10% CCK-8, and the cells were incubated at 37°C for 2 h. The absorbance at 450 nm was measured using a 96-well microplate reader (Bio-Rad Laboratories, Hercules, CA, United States).

### Quantitative Real-Time PCR

Bone marrow-derived macrophages were cultured in 6-well plates with or without 5 μM KP-A038 in osteoclast-inducing media. Total RNA was extracted using TRI-solution (Bioscience, Seoul, South Korea) according to the manufacturer’s instructions. cDNA was synthesized using SuperScript II Reverse Transcriptase (Invitrogen, Carlsbad, CA, United States). Real-time PCR was performed using a LightCycler 1.5 real-time PCR system (Roche Diagnostics, Basel, Switzerland) and the SYBR Premix Ex Taq (Takara Bio Inc., Shiga, Japan) (Ihn et al., 2018). The primer sequences used in real-time PCR analysis were: *Acp5*, 5′-TCCCCAATGCCCCATTC-3′ and 5′-CGGTTCTGGCGATCTCTTTG-3′; *Ctsk*, 5′-GGCTGTGGAG GCGGCTAT-3′ and 5′-AGAGTCAATGCCTCCGTTCTG-3′; *Mmp9*, 5′-AAAGACCTGAAAACCTCCAACCT-3′ and 5′-GCCCGGGTGTAACCATAGC-3′; *Dcstamp*, 5′-CTTC CGTGGGCCAGAAGTT-3′ and 5′-AGGCCAGTGC TGACTAGGATGA-3′; *Nfatc1*, 5′-ACCACCTTTCCGCAACCA-3′ and 5′-TTCCGTTTCCCGTTGCA-3′; *Irf8*, 5′-GA TCGAACAGATCGACAGCA-3′ and 5′-AGCACAGCGTAA CCTCGTCT-3′; *Bcl6*, 5′-ATGAGATTGCCCTGCATTTC-3′ and 5′-TTCTTCCAGTTGCAGGCTTT-3′; *Ifng*, 5′-TCAAGT GGCATAGATGTGGAAGAA-3′ and 5′-TGGCTCTGCAGGATT TTCATG-3′; *Prdm1*, 5′-TTCTTGTGTGGTATTGTCGGG ACTT-3′ and 5′-TTGGGGACACTCTTTGGGTAGAGTT-3′.

### Immunoblotting

Cells were lysed with RIPA buffer containing protease and phosphatase inhibitors. Equal volume of cell lysates (25 μg of protein) was loaded onto 10% sodium dodecyl sulfate-polyacrylamide gels, followed by transfer to nitrocellulose membranes (Whatman Inc., Florham Park, NJ, United States). The membranes were placed in a blocking solution [3% non-fat dry milk in TBS-T (25 mM Tris–HCl, pH 7.4, 150 mM NaCl, and 0.2% Tween 20)] for 1 h. After blocking, the membranes were incubated with specific primary antibodies (1:1000) at 4°C overnight. Following incubation with secondary antibodies, protein signals were detected using WesternBright ECL (Advansta, Menlo Park, CA, United States), and imaged with an X-ray film or by using chemiluminescence imager (Azure Biosystems, Inc., Dublin, CA, Unites States).

### Staining of Actin Rings

Bone marrow-derived macrophages grown on glass coverslips were cultured with 10 ng/mL M-CSF and 20 ng/mL RANKL in the presence or absence of 5 μM KP-A038. The cells were washed with phosphate buffered saline, fixed with 4% paraformaldehyde, and permeabilized with 0.1% Triton X-100. F-actin was stained with rhodamine conjugated phalloidin (Cytoskeleton, Denver, CO, United States), and 4’,6-diamidino-2-phenylindole dihydrochloride (Santa Cruz Biotechnology, Santa Cruz, CA, United States) was used for nuclei staining. The images were acquired using a BX51 Fluorescent Microscope (Olympus, Tokyo, Japan).

### Resorption Pit Assay

Bone marrow-derived macrophages were placed on bone slices (IDS Nordic Bioscience, Herlev, Denmark) and incubated with M-CSF (10 ng/mL) and RANKL (20 ng/mL) to induce differentiation of BMMs into multinucleated osteoclasts. After 3 days, the cells were treated with vehicle or 5 μM KP-A038 for 2 days. The cells removed with 1 N NaOH for 20 min, followed by staining with hematoxylin to identify the areas of resorption pits. The resorbed areas were measured using the i-Solution image analysis program (IMT i-Solution, Daejeon, South Korea).

### *In vivo* LPS-Induced Bone Loss

All animal experiments were approved by the Animal Care and Use Committee at Kyungpook National University and were conducted in accordance with the guidelines for the care and use of laboratory animals. To study the effect of KP-A038 on *in vivo* bone destruction, 8-week-old C57/B6L mice were intraperitoneally injected with KP-A038 (30 mg/kg) or vehicle daily for 9 days, and LPS (5 mg/kg) was intraperitoneally administered to the mice on days 2 and 6, as previously described (Ihn et al., 2015a). On day 10, the mice were euthanized by cervical dislocation under anesthesia with avertin. The femurs were isolated and fixed in 4% paraformaldehyde for 18 h.

### Micro-CT and Histomorphometric Analysis

The fixed femurs were scanned using high-resolution μCT (Skyscan 1272; Kontich, Belgium) with a source voltage of 60 kV, current of 166 μA, and resolution of 14 μm. Bone morphometric parameters, including bone volume per total volume (BV/TV), bone mineral density (BMD), trabecular separation (Tb. Sp.), and trabecular number (Tb. N.) were analyzed using CTAn software (Bruker; Kontich, Belgium). Micro-CT 3D images of trabecular bones in the distal femurs were generated using CTAn/CTVol software (Bruker; Kontich, Belgium).

For histology, the fixed femurs were decalcified in 12% EDTA and embedded in paraffin, and the histological tissue sections were stained with hematoxylin and eosin and TRAP.

### Statistics

Experiments were conducted three times, and all data are presented as mean±standard deviation (SD). Statistical significance was evaluated by the two-tailed Student’s *t*-test or one-way analysis of variance (ANOVA) with Tukey’s multiple comparison *post hoc* test. *p <* 0.05 or *p <* 0.01 was considered statistically significant.

## Results

### KP-A038 Prevents LPS-Induced Bone Resorption *in vivo*

**FIGURE 2:**
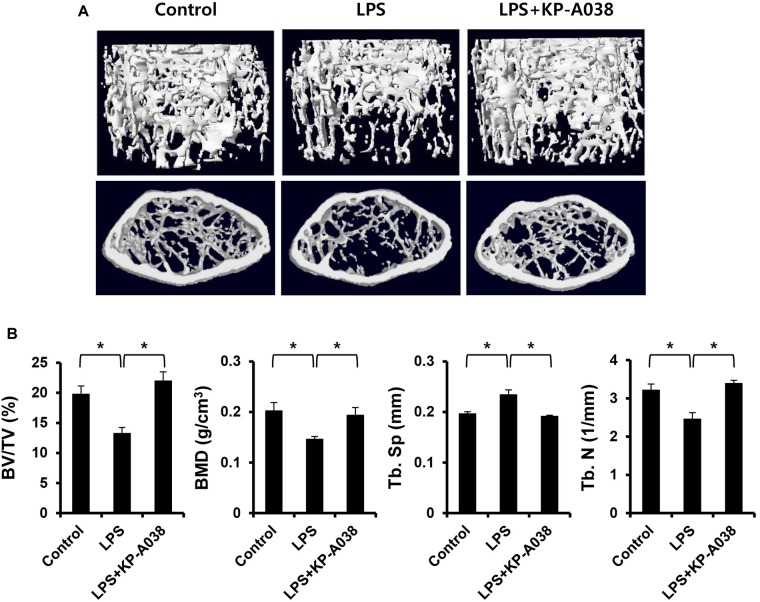
KP-A038 mitigated femoral bone loss induced by LPS *in vivo*. **(A)** Representative micro-CT images of distal femurs of three groups. **(B)** Quantification of bone volume per tissue volume (BV/TV), bone mineral density (BMD), trabecular separation (Tb. Sp), and trabecular number (Tb. N) from each group. *n* = 5 (10 legs) in each group. ^∗^*p* < 0.05.

**FIGURE 3:**
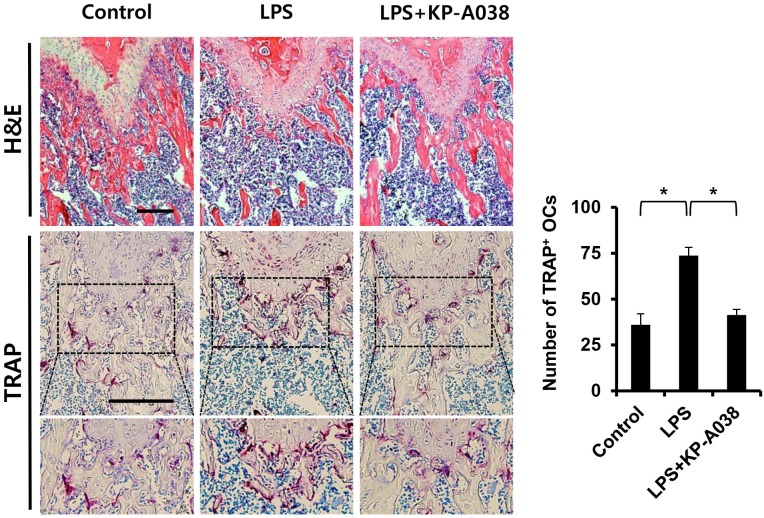
Histological analysis of the effect of KP-A038 on LPS-mediated bone destruction. Fixed femurs were decalcified and sectioned. The sections were stained with hematoxylin and eosin (upper panel) and tartrate-resistant acid phosphatase (TRAP, middle and lower panel). The number of osteoclasts was analyzed in the TRAP-stained slices (right graph). *n* = 5 (10 legs) in each group. ^∗^*p* < 0.05.

To examine the *in vivo* efficacy of KP-A038 in osteoclast formation and bone resorption, we used an LPS-induced bone erosion model. Mice were intraperitoneally administered with LPS on days 2 and 6 and received vehicle or KP-A038 daily for 9 days. Injection of KP-A038 alone did not cause adverse events, including death, abnormal behavior, sickness, and distress or change bone parameters (Supplementary Figure S2). Administration of LPS led to a reduction in trabecular bone mass of femurs, and this LPS-mediated bone loss was suppressed by co-injection with KP-A038 (Figure 2A). Three-dimensional morphometric analysis of distal femurs showed that BV/TV, BMD, and Tb. N were significantly reduced in LPS-treated group (Figure 2B). Such reduction in bone parameters was attenuated by KP-A038 treatment (Figure 2B). In correlation with μCT images and analysis of bone parameters, the histological sections stained with hematoxylin and eosin or TRAP showed that KP-A038 effectively suppressed LPS-induced osteoclast formation and subsequent bone loss *in vivo* (Figure 3).

### KP-A038 Inhibits RANKL-Induced Osteoclast Formation *in vitro*

**FIGURE 4:**
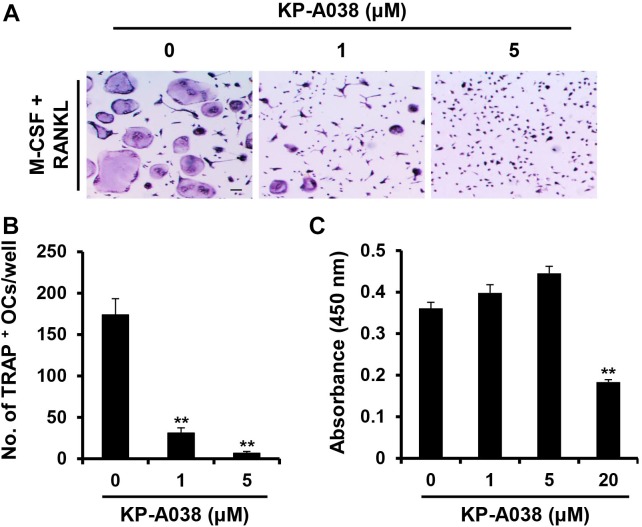
KP-A038 inhibited RANKL-mediated osteoclastogenesis without cytotoxicity. **(A)** BMMs were incubated in osteoclastogenic medium supplemented with M-CSF (10 ng/mL) and RANKL (20 ng/mL) in the presence of KP-A038 or vehicle. The cells were stained to evaluate TRAP activity. **(B)** The number of osteoclast-like cells was quantified. **(C)** BMMs were cultured with M-CSF (10 ng/mL) and various doses of KP-A038 for 3 days. Cell viability was assessed by CCK-8 assay. ^∗∗^*p* < 0.01.

To clarify whether KP-A038 affects the viability of osteoclast progenitors, CCK-8 assay was performed using primary BMMs cultured with M-CSF and various doses of KP-A038 for 3 days. As shown in Figure 4C, KP-A038 at concentrations of up to 5 μM did not decrease the rate of proliferation and survival of BMMs. Hence, 5 μM KP-A038 was chosen for subsequent *in vitro* studies. As primary BMMs are capable of differentiating into multinucleated osteoclasts in response to M-CSF and RANKL, we first examined the effect of KP-A038 on osteoclast formation in BMMs. Primary cultured BMMs were treated with KP-A038 (1 μM or 5 μM) in osteoclast-inducing media supplemented with M-CSF and RANKL. RANKL promoted the formation of osteoclasts (TRAP-positive MNCs) from progenitors (BMMs) in the vehicle-treated control group, whereas treatment with KP-A038 markedly suppressed osteoclast formation in a concentration-dependent manner (Figure 4A). In the presence of 5 μM KP-A038, the number of TRAP-positive MNCs was reduced by 96% (Figure 4B).

### KP-A038 Attenuates the Expression of Osteoclast-Specific Markers as Well as the Formation of Actin Rings

**FIGURE 5:**
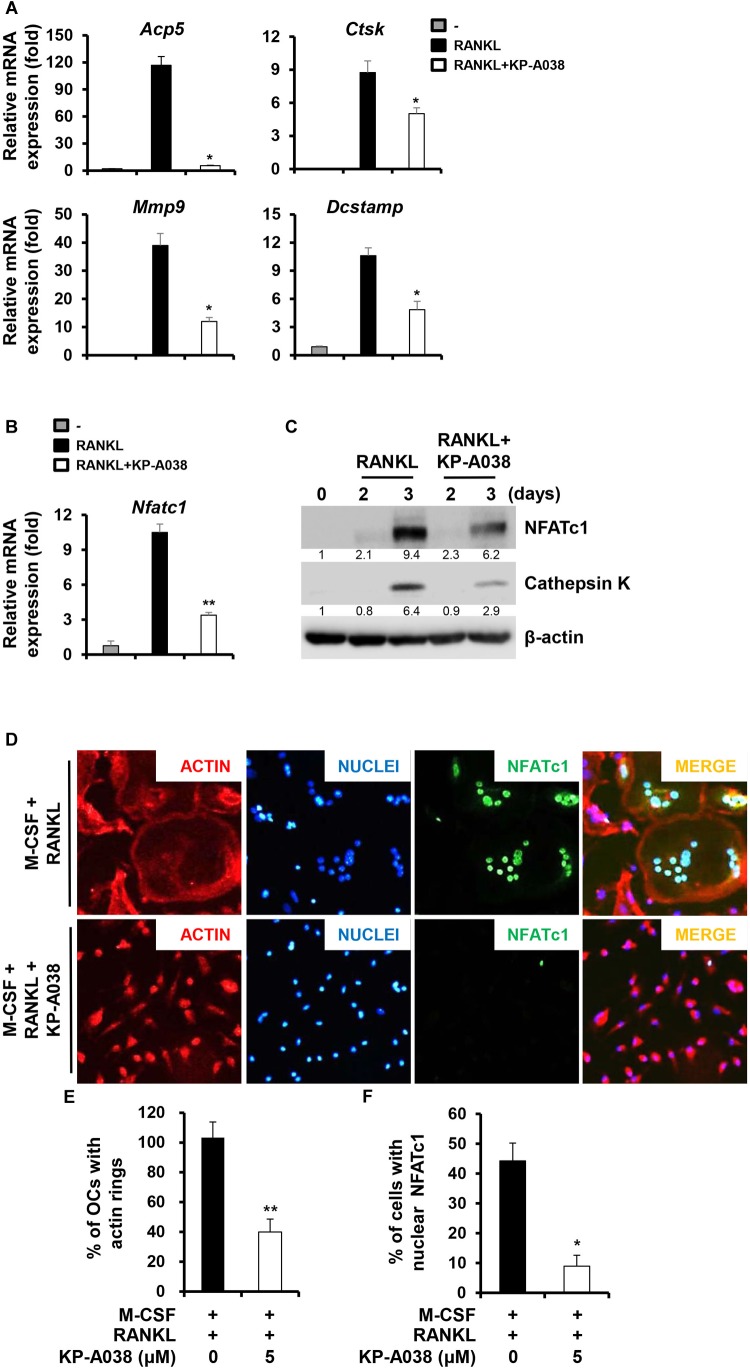
KP-A038 suppressed the expression of osteoclast-specific markers and the formation of actin rings. **(A–C)** BMMs were incubated in osteoclastogenic medium with KP-A038 (5 μM) or vehicle for the indicated times. The mRNA **(A,B)** and protein **(C)** expression levels of markers were evaluated by real-time PCR and western blot analysis, respectively. **(D)** BMMs were seeded on glass coverslips and cultured with M-CSF (10 ng/mL) and RANKL (20 ng/mL) in the presence or absence of 5 μM KP-A038. Cells were fixed, and F-actin structures and nuclei were visualized by staining with rhodamine-conjugated phalloidin and 4’,6-diamidino-2-phenylindole dihydrochloride, respectively. Quantification of the percentage of **(E)** osteoclasts with actin rings and **(F)** cells with nuclear NFATc1. ^∗^*p* < 0.05, ^∗∗^*p* < 0.01.

We next evaluated the mRNA and protein expression levels of osteoclast-specific markers during RANKL-induced osteoclast differentiation via real-time PCR and western blotting to further determine its inhibitory role in osteoclastogenesis. Consistent with the decreased osteoclast formation, treatment with KP-A038 (5 μM) downregulated the mRNA levels of *Acp5*, *Ctsk*, *Dcstamp*, *Mmp9*, and *Nfatc1*, which are required for osteoclast formation and/or bone resorption (Figure 5A,B). In addition, the induction of both cathepsin K (Ctsk) and NFATc1 proteins and the nuclear expression of NFATc1 were decreased by treatment with 5 μM KP-A038 (Figure 5C,F).

An essential step in the generation of multinucleated osteoclasts is cell-cell fusion, and DC-STAMP is a key regulator of this process (Yagi et al., 2005). In accordance with the decreased induction of *Dcstamp*, we found that the number of multinucleated giant cells with actin ring structure was significantly decreased by KP-A038 treatment (Figure 5D,E).

### KP-A038 Impairs the Bone-Resorbing Function of Osteoclasts

**FIGURE 6:**
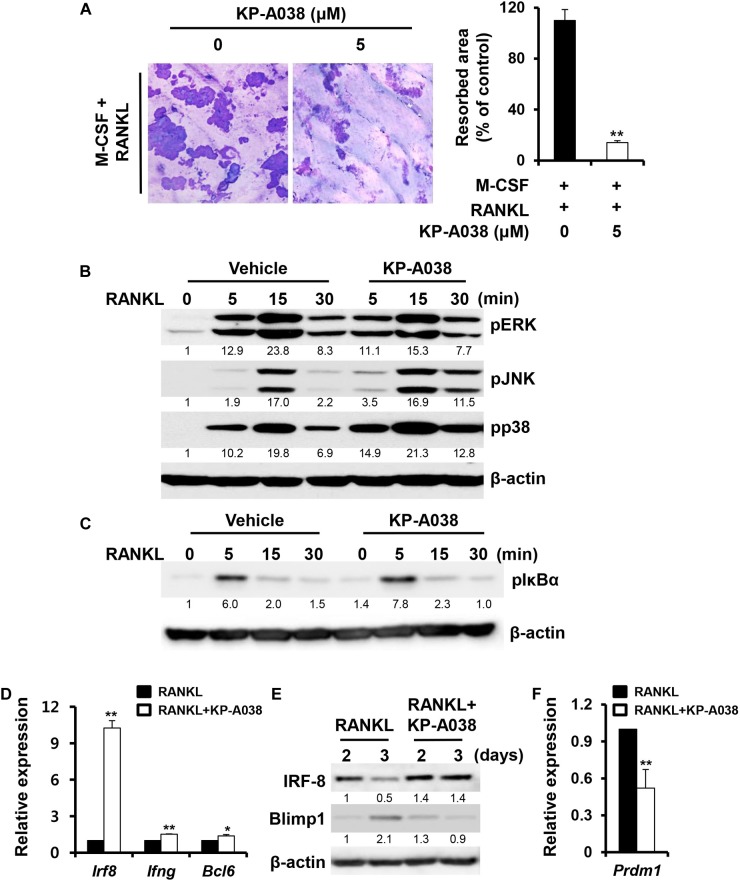
KP-A038 attenuated osteoclastic resorption activity and modulated the expression of negative regulators of osteoclastogenesis. **(A)** BMMs were seeded on bone slices and incubated in osteoclast- inducing medium. After 3 days, vehicle or 5 μM KP-A038 was added to the cell culture for 24 h and the resorptive areas were quantified using the i-Solution program (right graph). **(B,C)** BMMs were pretreated with vehicle or KP-A038 (5 μM) for 1 h followed by stimulation with RANKL (50 ng/mL) for indicated periods. Phosphorylation of ERK, JNK, p38, and IκBα was assessed by western blot analysis. β-actin was used as the loading control. **(D,F)** The mRNA expression of *Irf8*, *Ifng*, *Bcl6*, and *Prdm1* was analyzed by real-time PCR. **(E)** BMMs were cultured with M-CSF (10 ng/mL) and RANKL (20 ng/mL) in the presence or absence of 5 μM KP-A038 for indicated times. The protein expression of IRF-8 and Blimp1 was evaluated by immunoblotting. ^∗^*p* < 0.05, ^∗∗^*p* < 0.01.

We next examined if KP-A038 affects the bone-resorbing activity of osteoclasts. BMMs were plated on bone slices in osteoclastogenic media to generate multinucleated osteoclast-like cells. Then, the cells were treated with vehicle or 5 μM KP-A038 in osteoclast-inducing media for 2 days. Analysis of resorption pit showed that KP-A038 treatment significantly reduced the formation of resorption pits compared to vehicle treatment, which resulted in formation of larger resorbed areas. Addition of KP-A038 resulted in an 87% reduction of the resorbed area (Figure 6A), thereby suggesting that KP-A038 directly attenuates the bone-resorbing function of osteoclasts.

### KP-A038 Inhibits the Repression of Negative Regulators of Osteoclast Differentiation

To understand the molecular mechanism of the inhibitory action of KP-A038 in osteoclast differentiation and function, the effect of KP-A038 on RANKL-stimulated MAPK and NF-κB signaling pathways was examined. Serum-starved BMMs were stimulated with RANKL after pretreatment with vehicle or 5 μM KP-A038 for 1 h. Stimulation with RANKL led to activation of MAPKs (ERK, p38, and JNK) and IκBα within 15 min in control cells, and pretreatment with KP-A038 had no effect on the phosphorylation levels of MAPKs and IκBα, indicating that KP-A038 does not affect the MAPK and NF-κB signaling pathways (Figure 6B,C).

RANKL-RANK signaling pathways ultimately converge on the induction of NFATc1, which is the main transcriptional regulator of osteoclastogenesis, and its activity is negatively regulated by IRF-8 and Bcl6 during osteoclast differentiation (Zhao et al., 2009; Miyauchi et al., 2010). Hence, we examined the expression levels of anti-osteoclastogenic genes, such as *Irf8*, *Ifng*, and *Bcl6*. While the mRNA expression of *Irf8*, *Ifng*, and *Bcl6* was downregulated by RANKL stimulation in vehicle-treated control group, such downregulation was blocked in the presence of 5 μM KP-A038 (Figure 6D). As shown in Figure 6E, IRF-8 protein expression was similarly upregulated in a time-dependent manner. Blimp1 (encoded by *Prdm1*) is known to function as a transcriptional repressor of IRF-8 and Bcl6, and Blimp1-mediated *Irf8,* and *Bcl6* suppression is critical for osteoclast differentiation (Zhao et al., 2009; Miyauchi et al., 2010). Consistent with increased *Irf8* and *Bcl6* expression, Blimp1 mRNA and protein levels were reduced in the presence of KP-A038 (Figure 6E,F).

## Discussion

Although the bone is remodeled through coordinated destruction and deposition, and the remodeling process is strictly regulated, abnormal bone remodeling can arise due to various causes, inducing skeletal diseases ranging from mild to severe. In particular, enhanced osteoclastogenesis and resorbing activity of mature osteoclasts causes destructive bone diseases. Therefore, the suppression of osteoclast differentiation and bone-resorbing function of mature osteoclasts is an important aspect for preventing and treating osteolytic diseases. In the current study, we observed that KP-A038, a novel imidazole compound, possessed anti-osteoclastogenic activity via downregulation of NFATc1 and modulation of the expression of negative regulators of osteoclast differentiation. Consistent with the suppression of osteoclast differentiation *in vitro*, results from the *in vivo* LPS-induced bone destruction study demonstrated that KP-A038 protected against bone loss by attenuation of osteoclast formation.

RANKL-RANK signaling ultimately causes the induction of NFATc1, which acts as a key transcription factor of osteoclastogenesis. Even though RANKL signaling is required for osteoclast differentiation, embryonic stem cells lacking NFATc1 fail to undergo osteoclast differentiation even in the presence of RANKL (Takayanagi et al., 2002). Furthermore, ectopic expression of NFATc1 in BMMs leads to osteoclast differentiation without RANKL signaling (Takayanagi et al., 2002). Overexpression of NFATc1 in osteoclast precursors lacking c-Fos, a pivotal regulator of early activation of NFATc1, rescues osteoclast differentiation *in vitro* (Matsuo et al., 2004). Aliprantis et al. (2008) reported that osteoclast-specific NFATc1 deficiency causes osteopetrosis due to impaired osteoclast differentiation. These *in vivo* and *in vitro* studies have established the essential role of NFATc1 in osteoclast formation and function. We demonstrated that KP-A038 treatment markedly downregulated both mRNA and protein levels of NFATc1, resulting in inhibition of osteoclastogenesis (Figure 5B,C). As expected, downregulation of NFATc1 by KP-A038 treatment attenuated the expression of direct transcriptional target genes, including *Acp5*, *Ctsk*, *Dcstamp*, and *Mmp9* (Figure 5A). Among them, DC-STAMP is essential for cell-to-cell fusion, which is a critical process to generate MNCs and reorganize the actin cytoskeleton during osteoclast differentiation (Yagi et al., 2005). Due to the reduced *Dcstamp* levels, KP-A038 treatment suppressed the formation of actin rings (Figure 5D). Our study suggested that KP-A038 might target NFATc1 leading to inhibition of osteoclast differentiation and impairment of cellular fusion.

The activation of signaling molecules, like MAPKs and NF-κB involved in the RANKL/RANK signaling pathway, is an early cellular event of osteoclast differentiation, eventually leading to the induction NFATc1 (Iotsova et al., 1997; Mizukami et al., 2002). To understand the molecular mechanisms of inhibition of osteoclast differentiation and bone-resorbing function, we analyzed the extent of phosphorylation of the signaling molecules. Although our studies showed that KP-A038 definitely reduced the expression of NFATc1 and its target genes, KP-A038 treatment did not inhibit the activation of RANKL-mediated MAPKs and NF-κB (Figure 6B,C). Previously we screened and identified several chemicals from our in-house chemical library that exerted inhibitory effects on osteoclast differentiation and bone resorption. Although those compounds exhibited anti-osteoclastogenic and antiresorptive activities with a similar inhibitory concentration, the inhibitory mechanism and chemical structure of the compounds were quite distinct one another (Ihn et al., 2015a,b, 2017, 2018). In particular, unlike our previous compounds, KP-A038 did not inhibit an early RANKL-RANK signaling pathways (Figure 6B,C), suggesting different mode of action of KP-A038 from our previous chemicals. RANKL/RANK signaling has also been shown to downregulate various transcriptional repressors, including MafB, IRF-8, and Bcl6, which act as anti-osteoclastogenic factors via downregulating NFATc1 expression (Kim et al., 2007; Zhao et al., 2009; Miyauchi et al., 2010). Nishikawa et al. found that a transcriptional repressor of the negative regulators of NFATc1, Blimp 1 (*Prdm1*), is induced by RANKL stimulation, and Blimp 1-mediated suppression of anti-osteoclastogenic factors is necessary for osteoclastogenesis (Nishikawa et al., 2010). Therefore, forced expression of the negative regulators leads to impaired osteoclast differentiation. Among them, IRF-8 suppresses NFATc1 autoamplification and its transcriptional activity, and Bcl6 inhibits the expression of NFATc1 and its target genes associated with osteoclastogenesis and bone resorption (Zhao and Ivashkiv, 2011). In our study, KP-A038 prevented the downregulation of negative regulators of osteoclastogenesis, and in particular, the mRNA and protein expression of IRF-8 was strongly induced in the presence of KP-A038 (Figure 6D,E). Consistently, KP-A038 suppressed both mRNA and protein levels of Blimp1 (Prdm1) (Figure 6E,F). These results indicated that the inhibitory effect of KP-A038 on osteoclastogenesis might be in part mediated by the failure of repression of negative regulators. Further studies would be necessary to identify the primary target (s) for KP-A038.

The major role of osteoclasts is to break down the bone matrix, which is termed as bone resorption. Various antiresorptive drugs, such as bisphosphonates and selective estrogen receptor modulators have been clinically used for preventing further loss of bone density (Migliaccio et al., 2007). Therefore, we evaluated the effect of KP-A038 on bone-resorbing function by resorption pit assay. KP-A038, at a concentration that inhibited osteoclast differentiation, considerably suppressed the ability of osteoclasts to degrade the calcified bone matrix (Figure 6A), indicating that KP-A038 exhibits not only anti-osteoclastogenic but also antiresorptive activity.

Lipopolysaccharide is a structural constituent of the outer membrane of gram-negative bacteria and it functions as a potent stimulator of bone loss. The production of inflammatory cytokines in response to LPS is increased, which directly and indirectly contributes to stimulation of osteoclastogenesis and inflammatory bone loss (Wada et al., 2004; Islam et al., 2007). Especially, alveolar bone destruction mediated by inflammatory responses in the oral cavity can lead to tooth loss (Pihlstrom et al., 2005). For that reason, it is critical to prevent the balance of two opposing activities (bone formation and bone resorption) from breaking for maintenance and regeneration of alveolar bone and supporting tissue (Larsson et al., 2016). Consistent with its inhibitory effect on osteoclast differentiation and function *in vitro*, KP-A038 attenuated the femoral bone destruction induced by LPS (Figure 2). Administration of KP-A038 decreased the numbers of TRAP-positive osteoclasts as well as bone resorption *in vivo* (Figure 3), indicating that KP-A038 may have the potential to not only protect from the risk of inflammatory bone loss in periodontitis but also to contribute to bone regeneration in the oral cavity.

## Conclusion

Developing alternative agents that modulate excessive osteoclast formation and bone resorption is an important and urgent task. The results of this study demonstrated that KP-A038 exhibited anti-osteoclastogenic and antiresorptive properties by inhibiting the induction of NFATc1 via modulating the expression of negative regulators of osteoclastogenesis. Furthermore, KP-A038 protected against LPS-induced femoral bone loss *in vivo*. Our results suggest that KP-A038 might serve as a novel antiresorptive agent for osteoclast-related diseases.

## Ethics Statement

All animal experiments were approved by the Animal Care and Use Committee at Kyungpook National University and were conducted in accordance with the guidelines for the care and use of laboratory animals.

## Author Contributions

HI, TL, and EP designed the research and wrote the manuscript. HI and DL performed the research and collected the data. J-SB, S-HK, IJ, YB, and H-IS analyzed the data and clarified the manuscript.

## Conflict of Interest Statement

The authors declare that the research was conducted in the absence of any commercial or financial relationships that could be construed as a potential conflict of interest.
