# Supplementary material for: Inhibitory Effect of KP-A038 on Osteoclastogenesis and Inflammatory Bone Loss Is Associated With Downregulation of Blimp1
Source: Front Pharmacol. 2019 Apr 10;10:367. doi: 10.3389/fphar.2019.00367 (PMC6467953; doi:10.3389/fphar.2019.00367)
Supplement: Supplementary file 1 [file Data_Sheet_1.PDF]

Supplementary Material

Supplementary Figure 1.

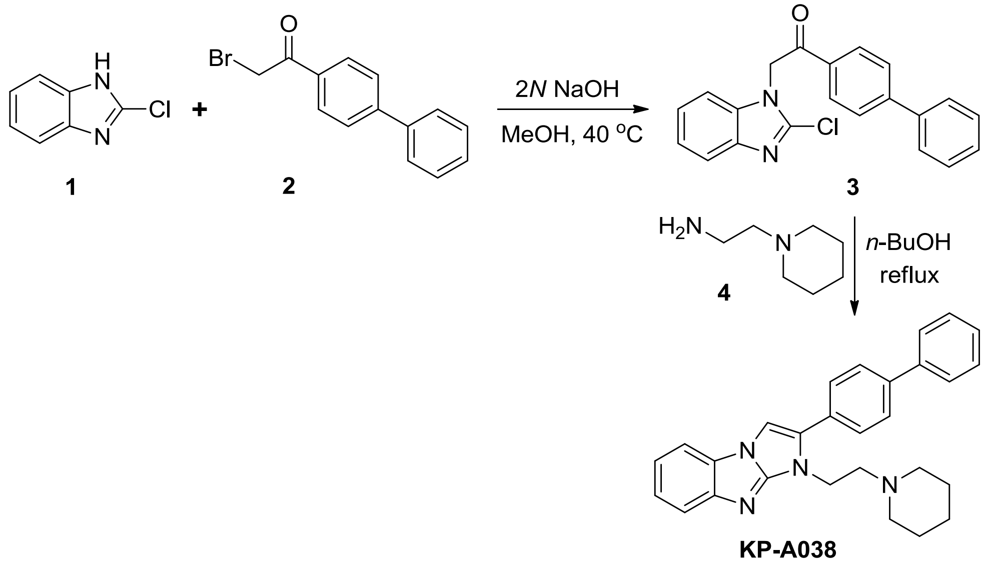

Supplementary Figure 1. Synthesis of 2-([1,1'-biphenyl]-4-yl)-1-(2-(piperidin-1-yl)ethyl)-1*H*-benzo[*d*]imidazo[1,2-a]imidazole (KP-A038).

A solution of 2-chlorobenzimidazole (**1**) (500 mg, 3.2 mmol), 1-([1,1'-biphenyl]-4-yl)-2-bromoethanone (**2**) (986 mg, 3.6 mmol), and NaOH (144 mg, 3.5 mmol) in 50% aqueous methanol was heated to 40 °C for 1 h. After cooling to room temperature, the reaction mixture was extracted with EtOAc (3×30 mL). The combined organic layers were dried over $MgSO_4$ and filtered. After concentration, the crude product was triturated with 25% EtOAc in hexane to give the 1-([1,1'-biphenyl]-4-yl)-2-(2-chloro-1*H*-benzo[*d*]imidazol-1-yl)ethanone (**3**) (915 mg, 83%) as a white solid. To a solution of **3** (200 mg, 0.576 mmol) in *n*-butanol (10 mL) was added 1-(2-aminoethyl)piperidine (**4**) (0.14 mL, 0.96 mmol). The reaction mixture was reflux for 15 h. After cooling to room temperature, the reaction mixture was concentrated under reduced pressure, and then the residue was purified by silica gel column chromatography to give the desired **KP-A038** (172 mg, 71%) as a white solid: ^{1}H NMR (500 MHz, $CDCl_3$) δ 1.35 (m, 2H), 1.43 (m, 4H), 2.32 (m, 4H), 2.79 (t, *J* = 6.5 Hz, 2H), 4.24 (t, *J* = 6.6 Hz, 2H), 7.14 (m 1H), 7.28 (s, 1H), 7.30 (m, 1H), 7.40 (m, 1H), 7.49 (m, 2H), 7.59 (d, *J* = 7.6 Hz, 2H),7.64-7.67 (m, 4H), 7.71-7.74 (m, 3H); LC-MS (ESI) *m/z* 421 ($[M+1]^+$).

Supplementary Figure 2.

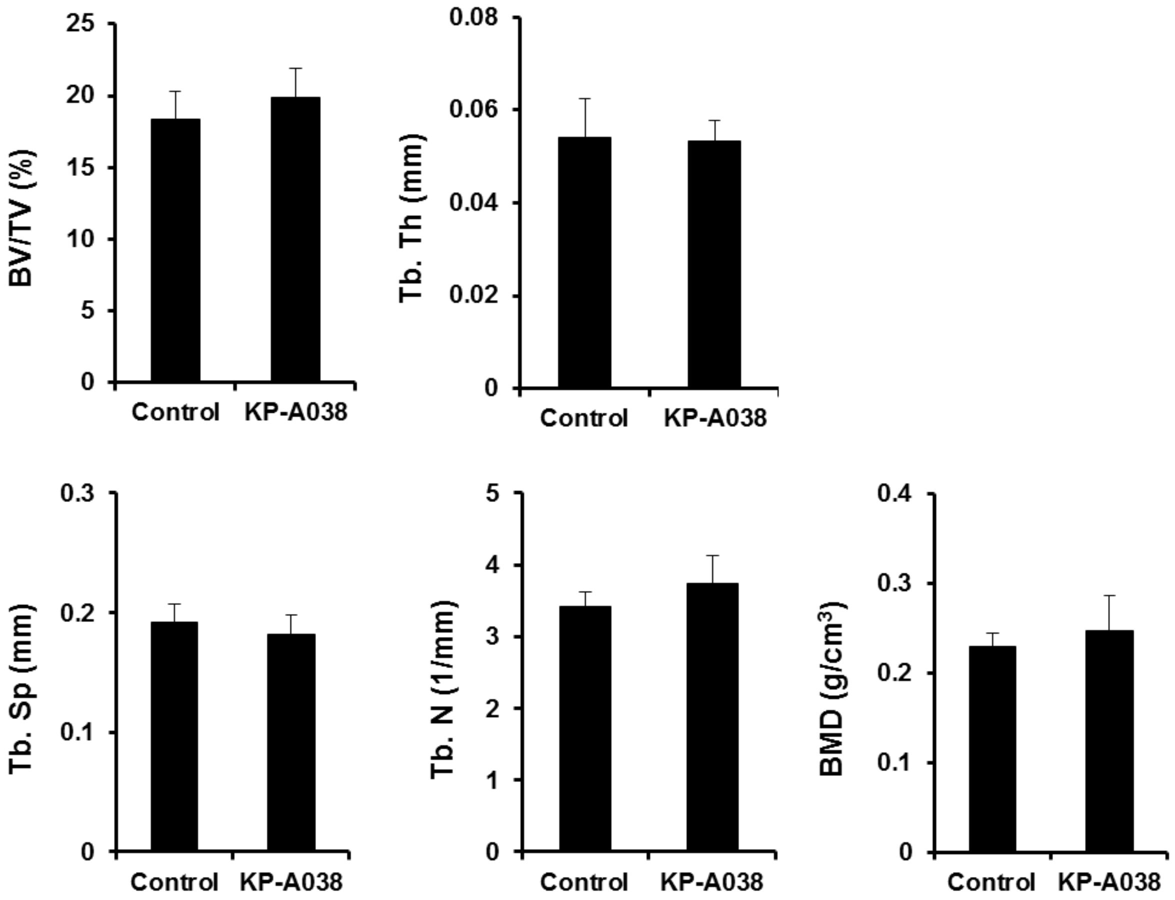

Supplementary Figure 2. Effect of KP-A038 on trabecular bone quality. Mice treated with vehicle- or KP-A038 (30 mg/kg) for 9 days were sacrificed, and bone volume per tissue volume (BV/TV), bone mineral density (BMD), trabecular separation (Tb. Sp), and trabecular number (Tb. N) were analyzed using CTAn software. n = 4 in each group.
